# Biological Function of PD-L2 and Correlation With Overall Survival in Type II Endometrial Cancer

**DOI:** 10.3389/fonc.2020.538064

**Published:** 2020-10-28

**Authors:** Oliviero Marinelli, Daniela Annibali, Maria Beatrice Morelli, Laura Zeppa, Sandra Tuyaerts, Cristina Aguzzi, Consuelo Amantini, Federica Maggi, Benedetta Ferretti, Giorgio Santoni, Frédéric Amant, Massimo Nabissi

**Affiliations:** ^1^School of Pharmacy, Experimental Medicine Section, University of Camerino, Camerino, Italy; ^2^School of Biosciences and Veterinary Medicine, Experimental Medicine Section, University of Camerino, Camerino, Italy; ^3^Gynecological Oncology, Oncology Department and LKI Leuven Cancer Institute, KU Leuven-University of Leuven, Leuven, Belgium; ^4^Department of Molecular Medicine, Sapienza University, Rome, Italy; ^5^Medical Oncology Division, San Severino Hospital, Macerata, Italy; ^6^Centre for Gynecologic Oncology Amsterdam (CGOA) Antoni Van Leeuwenhoek-Netherlands Cancer Institute (AvL-NKI) and University Medical Centra (UMC), Amsterdam, Netherlands; ^7^Integrative Therapy Discovery Lab, University of Camerino, Camerino, Italy

**Keywords:** programmed death ligand 2, endometrial cancer, immune checkpoint, overall survival, chemoresistance

## Abstract

In cancer, upregulation of coinhibitory B7 ligands has been associated with immune evasion. So far, anti-programmed death-1 (PD-1) and anti-PD-ligand 1 (PD-L1) antibodies have been used in immuno-oncology, with promising outcomes; however, it is still needed to identify other markers, especially for endometrial cancer (EC). EC is a gynecological malignancy historically classified into two types: type I, with mostly estrogen-dependent endometrioid diseases, and the most aggressive type II, including mainly estrogen-independent and non-endometrioid tumors. PD ligand-2 (PD-L2) is known as the second ligand of the PD-1 receptor and, upon its binding, contributes to T-cell exhaustion. Up to now, very few information are available about PD-L2 in cancers, and no data have been reported for EC. The aim of this work was to characterize the PD-L1 and PD-L2 ligand expression profile in EC cell lines, focusing the attention on the biological role of PD-L2 and its prognostic impact in human type II EC biopsies. Using *in silico* analysis of TCGA data, we performed a molecular profiling in a cohort of 506 patients, both types I and II, and PD-1 ligands expression was also analyzed in different primary human EC cell lines. Moreover, PD-L2 staining was evaluated in a cohort of human type II EC samples and correlated with the overall survival (OS), progression-free survival (PFS), and additional clinicopathological data. From the *in silico* analysis, PD-L2 was more expressed than PD-L1 in EC cell lines. PD-L2 was found highly expressed in 64.44% of tumor specimens, predominantly in the serous subtype, in both stromal and epithelial components, while in peritumoral and normal tissues it was predominantly moderate or low. *In vitro*, we investigated the cell autonomous role of PD-L2 in controlling cell survival, migration, and chemoresistance.

## Introduction

In developed countries, endometrial cancer (EC) is the most commonly diagnosed gynecological malignancy (1). Based on epidemiology, conventional histopathology and clinical behavior, EC is divided into two subtypes: estrogen-dependent endometrioid type I and estrogen-independent non-endometrioid type II (2). Endometrioid type I EC arises from hyperplastic tissue and is characterized by mutations of KRAS, β catenin, loss of PTEN expression, and microsatellite instability (MSI) (1). Non-endometrioid type II EC is less common but associated with an aggressive behavior and comprises only 3 to 10% of ECs but accounting for 39–44% of EC deaths (3). This group includes different non-endometrioid histological subtypes (e.g., serous or clear cell adenocarcinomas), and it develops from an atrophic endometrium. They are poorly differentiated, aggressive, and associated with a poor prognosis (1, 2). TP53 mutations occur in around 90% of serous adenocarcinomas, and these tumors are also characterized by aneuploidy, HER2 overexpression, p16 inactivation, and reduced E cadherin expression (1). Recently, “The Cancer Genome Atlas” has reframed type I/II distinction into four molecular categories: POLE-ultra mutated, MSI hypermutated, microsatellite stable (MSS), marked by endometrioid histology, and DNA copy number high marked by serous-like histology (4). Hysterectomy is indicated for early stage EC, while adjuvant treatments (hormonal therapy and combinations of radiation and chemotherapy) are suggested for higher stage EC, and promising results are obtained with triplet therapy with paclitaxel, cisplatin, and doxorubicin (TAP), but it is associated with significant toxicity. Then, generally, a doublet of paclitaxel and carboplatin (TC) is preferred, but a phase III non-inferiority trial comparing TAP and TC showed no significant difference in overall survival (OS) (5). However, only limited options remain if the tumor metastasizes (2). Therefore, novel and more effective therapies for patients with advanced or recurrent disease are needed (5). Cancer immunotherapy is emerging as a promising component for cancer therapy. The most promising immunotherapeutic approach involves antibodies targeting the immune checkpoint inhibitor (ICI) molecules, as demonstrated in Hodgkin’s lymphoma in which programmed death 1 (PD-1, CD279) receptor blockage resulted in response rate of 87%, probably based on the molecular upregulation of PD-1 and its ligands (PD-Ls) pathways (6, 7). Regarding EC, the role for PD-1 blockade in POLE-mutated and MSI-high tumors is clear, but for other molecular subtypes of EC, many aspects remain controversial in preclinical and clinical studies (8, 9).

PD-L1 (B7-H1, CD274) and PD-L2 (PDCD1LG2, B7-DC, CD273) are immune co-signaling molecules belonging to the B7 family, and they are expressed in several cancer types (6, 10) and, also, in infiltrating immune cells (11, 12). However, the prognostic value of PD-1 ligands is still debated, and their role, when expressed in the tumor microenvironment, has not been fully elucidated yet (9). It is known that PD-L1 and PD-L2 interaction with PD-1 receptor contributes to the strong inhibition of T-cell antitumor effects, resulting in immune escape (10, 13, 14). Indeed, previous studies showed that PD-L1 exerts tumor crucial cell-intrinsic signals for pathogenesis, including epithelial–mesenchymal transition, autophagy, resistance against proapoptotic stimuli, regulation of glucose metabolism, and activation of tumor mTOR/AKT signaling, supporting cancer cell proliferation and survival (13, 15). PD-L1 blocking antibodies have been approved for clinical use (15), while current and ongoing studies are trying to improve clinical responses for EC (16). So far, compared with PD-L1, the functional role of PD-L2 in cancer cells has been scarcely investigated (14). However, in patients with solid cancer, a meta-analysis suggested that PD-L2 might be involved in promoting tumor metastasis and predicts unfavorable prognosis, mainly in hepatocellular carcinomas (17). To our knowledge, there is no relevant literature reporting the expression of PD-L2 in non-endometrioid EC and its tumor intrinsic signaling effects. So, in this study, we evaluated PD-L2 expression in EC cell lines and human non-endometrioid EC biopsies, correlating its expression with different clinical features. In addition, we explored the signaling mechanisms regulated by PD-L2 in EC cell lines.

## Materials and Methods

### Endometrial Cancer Cell Lines

Ishikawa and MFE-280, respectively, well and poorly differentiated type I cell lines, were purchased from Sigma Aldrich (Milan, Italy). Ishikawa cells were grown in EMEM medium (Lonza, Milan, Italy), supplemented with 5% fetal bovine serum (FBS), 2 mM/L of glutamine, 100 IU/ml of penicillin, and 100 mg of streptomycin. MFE-280 cells were grown in EMEM medium (Lonza, Milan, Italy), supplemented with 10% FBS, 2 mM/L of glutamine, 100 IU/ml of penicillin, and 100 mg of streptomycin. HEC-1A and the primary EC cell lines PCEM002, PCEM004a, and PCEM004b were established in the lab of Frédéric Amant (Department of Oncology, KU Leuven, Leuven, Belgium). HEC-1A moderately differentiated type I cells were grown in Mc Coy’s medium (Lonza, Milan, Italy), supplemented with 10% FBS, 100 IU/ml of penicillin, and 100 mg of streptomycin, while the primary cell lines were grown in RPMI1640, supplemented with 20% FBS, 2 mM/L of glutamine, 100 IU/ml of penicillin, and 100 mg of streptomycin. PCEM002 is a poorly differentiated type I cell line, while PCEM004a and PCEM004b are poorly differentiated mixed type I/II cell lines. Media were changed every 48 h until cells were 90% confluent. All cell lines were maintained at 37°C with 5% CO_2_ and 95% humidity.

### Reagents and Drugs

RNAs and protein lysate from healthy donors (CU0000000015 and CI0000009692) were purchased from OriGene (Rockville, MD, United States). Cisplatin, doxorubicin, and paclitaxel were purchased from Sigma-Aldrich (Milan, Italy).

### TCGA and cBioportal Database Analysis

The cBioPortal for Cancer Genomics is an open-access downloaded bio-database, providing visualization and analyzing tool for large-scale cancer genomics data sets^1^. Analysis of 506 sequenced EC samples from this database (PanCancer Atlas) was performed in order to evaluate PD-Ls expression, following the online instructions of cBioPortal database for Genetic Alteration, Mutation level, Clinical Attribute, and mRNA expression. Briefly, the cancer-specific TCGA datasets were selected followed by the selection of mRNA expression z-scores relative to all samples (log RNA Seq V2 RSEM), PD-L1, and PD-L2 gene symbols, in the specified columns. On submitting the query, the software shows all types of genomic alterations including somatic mutations, copy number change, and mRNA expression, in a concise graphical summary called oncoprint. Then, data were downloaded, and mRNA expression values were analyzed with GraphPad.

### RNA Isolation, Reverse Transcription, and Quantitative Real-Time Polymerase Chain Reaction

Total RNA from cell lines was extracted with the RNeasy Mini Kit (Qiagen, Milan, Italy), and cDNA was synthesized using the iScript Advanced cDNA Synthesis Kit for RT-qPCR (Bio-Rad, Segrate, Italy) according to the manufacturer’s instructions. Quantitative real-time polymerase chain reactions (qRT-PCR) were performed with QuantiTect Primer Assays for Programmed Cell Death 1 Ligand 2 (CD273, PD-L2), Programmed Cell Death 1 Ligand 1 (CD274, PD-L1), and glyceraldehyde-3-phosphate dehydrogenase (GAPDH) (Qiagen), using the iQ5 Multicolor Real-Time PCR Detection System (Bio-Rad). The PCR parameters were 10 min at 95°C followed by 40 cycles at 95°C for 15 s and 60°C for 40 s. The relative amount of target mRNA was calculated by the 2^−ΔΔCt^ method, using GAPDH as a housekeeping gene. All samples were assayed in triplicates in the same plate. Measurement of GAPDH levels was used to normalize mRNA contents, and target gene levels were calculated by the 2^−ΔΔCt^ method.

### Western Blot Analysis

Twenty micrograms of total protein lysates were separated on an SDS polyacrylamide gel, transferred onto Hybond-C extra membranes (GE Healthcare, Milan, Italy), blocked with 5% low-fat dry milk in PBS-Tween 20, immunoblotted with goat anti-CD274 (PD-L1, 0.5 μg/ml, R&D System, Minneapolis, MN, United States), mouse anti-CD273 (PD-L2, 1 μg/ml, R&D System), rabbit anti-pAKT (1:1.000, Cell Signaling Technology, Danvers, MA, United States), rabbit anti-AKT (1:1.000, Cell Signaling), mouse anti-pERK (1:2.000, Cell Signaling Technology, Danvers, MA, United States), rabbit anti-ERK (1:1.000, Cell Signaling Technology), and anti-glyceraldehydes-3-phosphate dehydrogenase (GAPDH, 1:8.000, OriGene) antibodies (Abs) for 1 h and then incubated with HRP-conjugated anti-mouse or anti-rabbit secondary Abs (1:2.000, Cell Signaling Technology) and with HRP-conjugated anti-goat secondary Ab (1:1.000, Cell Signaling Technology) for 1 h. Peroxidase activity was visualized with the LiteAblot^®^PLUS or TURBO (EuroClone, Milan, Italy) kit, and densitometric analysis was carried out by a Chemidoc using the Quantity One software (Bio-Rad).

### Patient Samples

After obtaining approval from the Medical Ethics Committee UZ/KU Leuven (protocol nr S61970, Dec 2018), 53 archived formalin-fixed, paraffin-embedded type II EC samples, along with clinical data, and 15 normal tissues (of which five peritumoral tissues) were retrieved from UZ Leuven Biobank, Belgium. The sample set included 29 serous tumors, 7 clear cell tumors, 17 mixed types I and II, 5 peritumoral tissues from patients with type II EC, and 10 healthy endometrial samples.

### Immunohistochemistry

Paraffin slides (4 μm) were heated for 3 to 4 h at 55°C, deparaffinized in toluol, and rinsed in ethanol. Tissues were incubated for 30 min in 0.5% H_2_O_2_ (Merck Millipore, Milan, Italy) in methanol, to block the endogenous peroxidases. For PD-L2 staining, after washing in TBS, epitopes were retrieved for 2 h at 90°C in Tris–EDTA (pH = 9). Tissues were cooled down slowly in TBS. After extensive washing, tissues were blocked with a solution of 2% BSA (Sigma-Aldrich), 1% milk powder, and 0.1% Tween-80 (Merck Millipore) in TBS, before antibody incubation. After removal of blocking solutions, tissues were incubated with mouse anti-CD273 (PD-L2, 1 μg/ml, R&D System) in TBS, overnight at 4°C. After washing, sections were incubated with anti-mouse-HRP (Dako, Milan, Italy) for 30 min and washed again. Stainings were visualized by 10-min incubation in 3,3’-diaminobenzidine (DAB, Sigma-Aldrich) +0.015% H_2_O_2_ in the dark. Mayer’s hematoxylin was used to stain nuclei, and tissues were dehydrated in propanol, dipped in xylene, and mounted. To ensure no staining was caused by non-specific binding of secondary/tertiary molecules, control slides without addition of primary antibody were used.

### Immunohistochemistry Scoring Method

All stainings were evaluated semiquantitatively, using the Allred score system, adding score for intensity (0 = absent, 1 = weak, 2 = moderate, and 3 = strong) and score for percentage of stained cells (0 = absent, 1 = less than 1%, 2 = 1–10%, 3 = 11–33%, 4 = 34–66%, and 5 = 67–100%), to a maximum score of 8 (18). Stainings were evaluated only in the cellular component where expression was expected. Tissues were considered with a high expression at a cutoff score of 6, corresponding to strong positivity in ≥ 11% of cells, moderate positivity in ≥ 34% of cells, or weak staining in ≥67% of cells. This cutoff was considered clinically relevant for therapeutic applications, as a targeted therapy, because it would be more effective when the target is expressed in a sufficient percentage of cells. Tissues with a value between 4 and 5 were classified as moderate. Photographs of representative cases were taken using the Axioskop microscope (MRc5, Zeiss, Jena, Germany) equipped with the ZEN 2.0 software.

### Confocal Laser Scanning Microscopy Analysis

Ishikawa cells were seeded on eight-well culture slide in fresh medium, fixed, and permeabilized using 2% and 4% of paraformaldehyde with 0.5% of Triton X-100 (Sigma-Aldrich) in PBS. After washing with PBS, cells were incubated with 10% of FBS and 0.1% of Tween-20 in PBS for 1 h at room temperature and stained with mouse anti-PD-L2 overnight at 4°C. Then, the slide was washed with 0.3% of Triton X-100 in PBS and incubated with Alexa Fluor 594-conjugated secondary Ab (Cell Signaling Technologies) for 1 h at 37°C. Nuclei were stained with DAPI. The slide was then analyzed with C2 Plus confocal laser scanning microscope (Nikon Instruments, Firenze, Italy). Optimized emission detection bandwidths were configured by Zeiss Zen control software. Images were processed using NIS Element Imaging Software (Nikon Instruments, Firenze, Italy).

### PD-L2 Overexpression

PCEM004b cells were seeded at a density of 4 × 10^5^ cells/ml and, after 12 h of incubation, transfection was achieved with 3 μl/ml of the TurboFectin Transfection Reagent (OriGene) and 1 μg/ml of pCMV empty (pCMV6) and pCMV6–PDCD1LG2 vectors (OriGene), according to the manufacturer’s instructions. The cells were harvested at 72 h post-transfection for analysis. Transfection efficiency was evaluated by Western blot analysis.

### PD-L2 Silencing

Small interfering RNAs (siRNAs) targeted to PD-L2 (siPDCD1LG2) and a control non-silencing siRNA (NC1) were purchased from Riboxx GmbH (Radebeul, Germany). Ishikawa cells were plated at a density of 1 × 10^5^ cells/ml. After overnight incubation, transfections were achieved with 80 μl/ml of the reagent riboxxFECT and 20 nM of siPDCD1LG2 or NC1 (negative control), according to the manufacturer’s instructions. Cells were harvested at 72 h post-transfection for analysis. The efficiency of silencing was evaluated by Western blot analysis.

### Wound Healing Assay

PCEM004b and Ishikawa cells, native and transfected/silenced for PD-L2, were plated on a 24-well plate at density of 4 × 10^4^ and 1.5 × 10^5^/ml, respectively. Confluent cells were scratched using 10-μl sterile pipette tips, and low serum medium was added, to minimize cell proliferation and prevent cell detachment. Images of wounded areas were taken at 0, 24, and 48 h. Image acquisition was carried out by a Leitz Fluovert FU (Leica Microsystems) microscope. Remaining wound areas were determined using NIH Image J software for calculation of the percentage of wound closure. Analyses were performed in triplicate.

### MTT Assay

EC cell lines (3 × 10^4^ cells/ml) were plated in 96-well plates, in a final volume of 100 μl/well. After 24 h, treatments or vehicles were added for 72 h. At least six replicates were used for each treatment. At the indicated time point, cell viability was assessed by adding 0.8 mg/ml of 3-[4,5-dimethylthiazol-2-yl]-2,5 diphenyl tetrazolium bromide (MTT, Sigma-Aldrich) to the media. After 3 h, the plates were centrifuged, the supernatant was removed, and the pellet was solubilized with 100 μl/well of DMSO. The absorbance of the samples against a background control (medium alone) was measured at 570 nm using an ELISA reader microliter plate (BioTek Instruments, Winooski, VT, United States).

### Statistical Analysis

The data presented represent the mean with standard deviation (SD) of at least three independent experiments. The statistical significance was determined by Student’s *t*-test and by one-way and two-way ANOVA with Bonferroni’s posttest; ^∗^*p* < 0.05. Patients were divided in three groups according to high, moderate, or low expression of protein target. The Kaplan–Meier (KM) method was also used for OS and progression-free survival (PFS) analysis. For univariate and multivariate analysis of significance, the log-rank test or Cox analysis was used (Graph Pad and XLSTAT). A value of ^∗^*p* < 0.05 was considered as statistically significant. The statistical analysis of IC_50_ levels was performed using Prism 5.0a (Graph Pad).

## Results

### PD-1 Ligands Expression in EC Samples From TCGA and in EC Cancer Cell Lines

**FIGURE 1:**
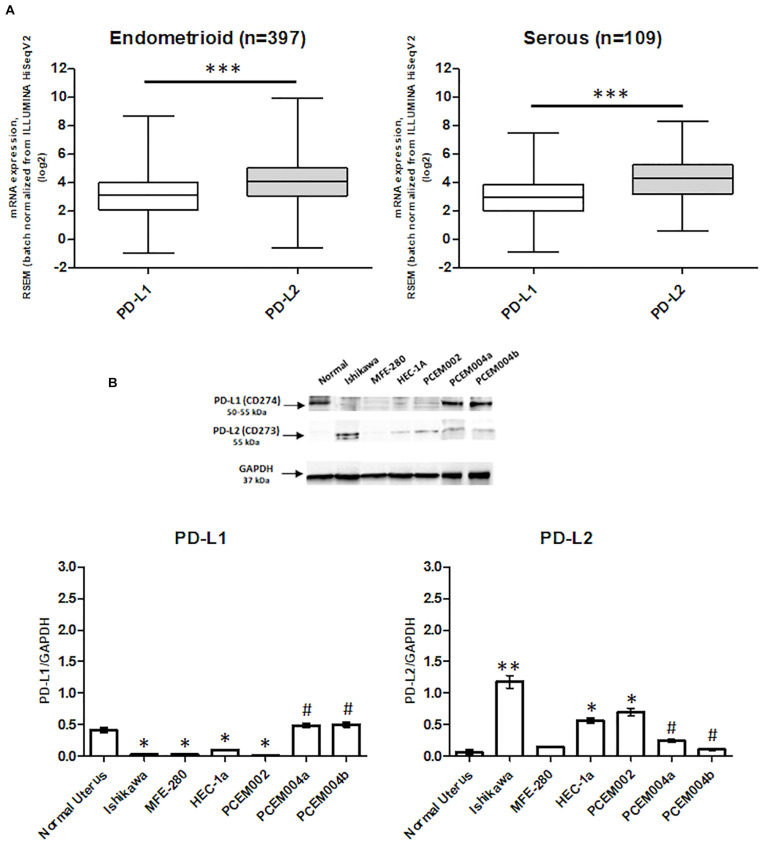
Programmed death-ligand 1 (PD-L1) and PD-L2 expression in endometrial cancer (EC). **(A)** The expression of PD-L1 and PD-L2 in EC patients. The mRNA expression (log2) of PD-L1 and PD-L2 in 506 EC samples, divided by 397 for type I and 109 for type II, from the TCGA database. ***p < 0.0001 PD-L2 vs PD-L1. **(B)** PD-L1 and PD-L2 protein expression was evaluated by Western blot in normal human uterus and six EC cell lines. PD-L1 and PD-L2 densitometry values were normalized to glyceraldehyde-3-phosphate dehydrogenase (GAPDH) used as loading control. Densitometric values shown are the mean ± SE of three separate experiments. *p < 0.05 vs normal control, ^#^*p* < 0.05 vs type I primary EC cell line PCEM002.

**FIGURE 2:**
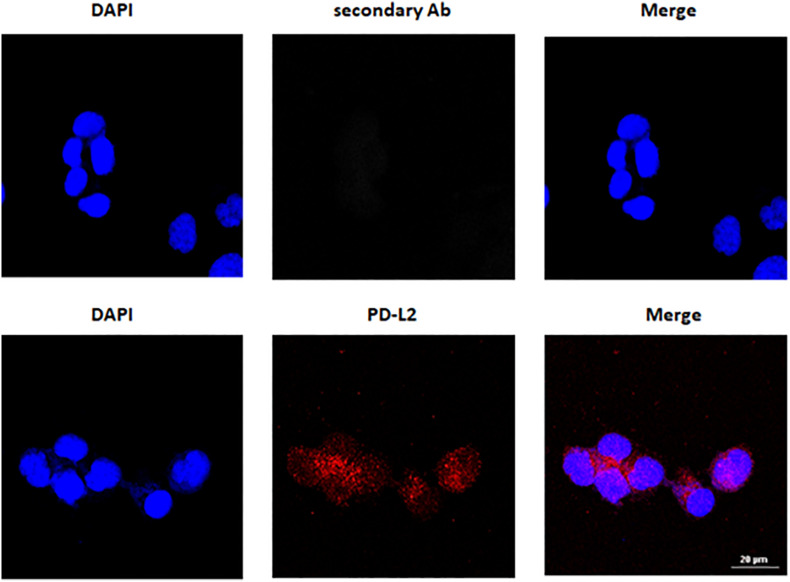
PD-L2 cytoplasmatic expression in Ishikawa cells. Cells were fixed, permeabilized, and stained with anti-human PD-L2 antibody (Ab) followed by Alexa Fluor-594 secondary Ab. 4’,6-Diamidino-2-phenylindole (DAPI) was used to counterstain the nuclei. Calibration bar: 20 μm.

Programmed death-1 ligand gene expression was assessed in 506 EC data samples from TCGA, queried with cBioportal (TCGA, PanCancer Atlas). Samples were divided into endometrioid (397 samples) and serous type (109 samples), and the mRNA levels were expressed in log2. PD-L2 mRNA expression was higher than PD-L1 (*p* < 0.0001), but no significant differences were observed between endometrioid and serous tumors (Figure 1A). The expression of PD-L1 and PD-L2 in normal uterine tissue obtained from a healthy donor and in six EC cell lines, two of which, PCEM004a and PCEM004b, are classified as mixed type I/II, was evaluated by RT-PCR (data not shown) and Western blot analysis. At the protein level, PD-L2 levels were significantly higher in most EC cell lines compared to normal uterus, while PD-L1 was expressed predominantly in both mixed type I/II PCEM004 cell lines but were not significantly different from the control. Furthermore, in three type I EC cell lines, Ishikawa, HEC-1a, and PCEM002, PD-L2 levels were higher than PD-L1 (Figure 1B). Since PD-L1 and PD-L2 expression profile in analyzed cell lines is in accordance with data from TCGA, our cell lines could be a representative model for PD-1 ligands in *in vitro* study. To determine cellular distribution, Ishikawa cells (expressing high levels of PD-L2) were analyzed by confocal laser scanning microscopy. Results show that PD-L2 has a punctuate distribution localized mainly in the cytoplasm (Figure 2).

### PD-L2 Expression in Human Biopsies of EC Type II

**FIGURE 3:**
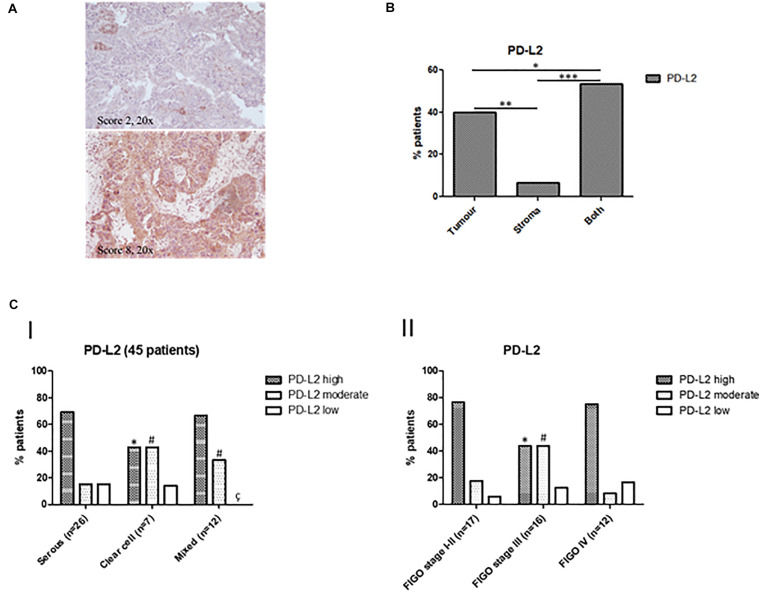
**(A)** Representative images of IHC stainings for PD-L2 in human EC biopsies. Stainings and scores of representative tumors. Pictures were taken at ×20 magnification (scale bar, 50 μm). **(B)** Percentages of patients classified according to PD-L2 distribution inside tumoral mass. **(C)** Classification of patients according to PD-L2 expression and clinicopathological characteristics. **(I)** Percentages of patients classified according to PD-L2 expression in EC, *p < 0.05 vs serous type PD-L2^*high*^, ^#^*p* < 0.05 vs serous type PD-L2^*moderate*^, ^ç^*p* < 0.05 vs serous type PD-L2^*low*^. **(II)** Percentage of patients classified according to PD-L2 expression and FIGO stage, *p < 0.05 vs FIGO stages **I–II** PD-L2^*high*^, ^#^*p* < 0.05 vs FIGO stages **I–II** PD-L2^*moderate*^.

On the basis of available literature and preliminary data obtained from our cell line models and PanCancer Atlas database, we investigated PD-L2 expression in a cohort of human EC type II. Its expression level was determined in a total of 51 samples, including serous, clear cell, mixed type, peritumoral tissues, and normal endometrium. Expression data are summarized in Table 1, divided for histological subgroups, FIGO stage, age, and tumor localization. Representative images of the stained samples classified according to the adopted scoring system, are shown (Figure 3A). Tissues were considered “high” at a score of 6 or higher, while “moderate” staining corresponds to a score of 4–5. PD-L2 was highly expressed in 64.44% of tumor specimens, while 24.44% of the samples stained moderate and 11.11% low or negative. PD-L2 was expressed predominantly in the epithelial component in 40% of the specimens and in both stromal and epithelial components in 53.33% of the samples (Figure 3B). The highest score was most frequently detected in serous type (69.23%) and mixed type (66.66%). Regarding peritumoral and normal tissues, PD-L2 was predominantly moderate or low (Figures 3C,I–II). The number of normal samples was low with respect to the initial selected cohort (10 samples) because the staining protocol used was incompatible with specimens rich in fat, according to H&E staining. Samples were classified according to the FIGO staging system, and the highest scores were detected for 76.47% stage I–II samples and for 75% stage IV samples (Figures 3C,III). An increased percentage of samples with strong positivity was detected in 69.23% of patients over 68 years (Figures 3C,IV). Taken together, our data suggest that PD-L2 could be a potential target for non-endometrioid EC, especially for serous and mixed subtypes, for low- and high-stage tumors.

**TABLE 1:** Expression of programmed death-ligand 2 (PD-L2) in endometrial cancer (EC), peritumoral tissue, and normal endometrium.

	PD-L2		
	High	Moderate	Low
Tumor	29/45 (64.44%)	11/45 (24.44%)	5/45 (11.11%)
Serous	18/26 (69.23%)	4/26 (15.38%)	4/26 (15.38%)
Clear cell	3/7 (42.85%)	3/7 (42.85%)	1/7 (14.28%)
Mixed	8/12 (66.66%)	4/12 (33.33%)	0/12 (0%)
Peritumoral tissue	1/4 (25%)	2/4 (50%)	1/4 (25%)
Normal endometrium	0/2 (0%)	1/2 (50%)	1/2 (50%)
**FIGO stage**			
Stage I–II	13/17 (76.47%)	3/17 (17.64%)	1/17 (5.88%)
Serous	7/9 (77.77%)	1/9 (11.11%)	1/9 (11.11%)
Clear cell	1/2 (50%)	1/2 (50%)	0/2 (0%)
Mixed	5/6 (83.33%)	1/6 (16.66%)	0/6 (0%)
Stage III	7/16 (43.75%)	7/16 (43.75%)	2/16 (12.5%)
Serous	5/10 (50%)	3/10 (30%)	2/10 (20%)
Clear cell	0/2 (0%)	2/2 (100%)	0/2 (0%)
Mixed	2/4 (50%)	2/4 (50%)	0/4 (0%)
Stage IV	9/12 (75%)	1/12 (8.33%)	2/12 (16.66%)
Serous	6/7 (85.71%)	0/7 (0%)	1/7 (14.28%)
Clear cell	2/3 (66.66%)	0/3 (0%)	1/3 (33.33%)
Mixed	1/2 (50%)	1/2 (50%)	0/2 (0%)
**Age**			
≤68	11/19 (57.89%)	5/19 (26.31%)	3/19 (15.79%)
>68	18/26 (69.23%)	6/26 (23.07%)	2/26 (7.69%)

*Percentages of samples according to different clinicopathological characteristics.*

### High Levels of PD-L2 Correlate With Poor Prognosis

**FIGURE 4:**
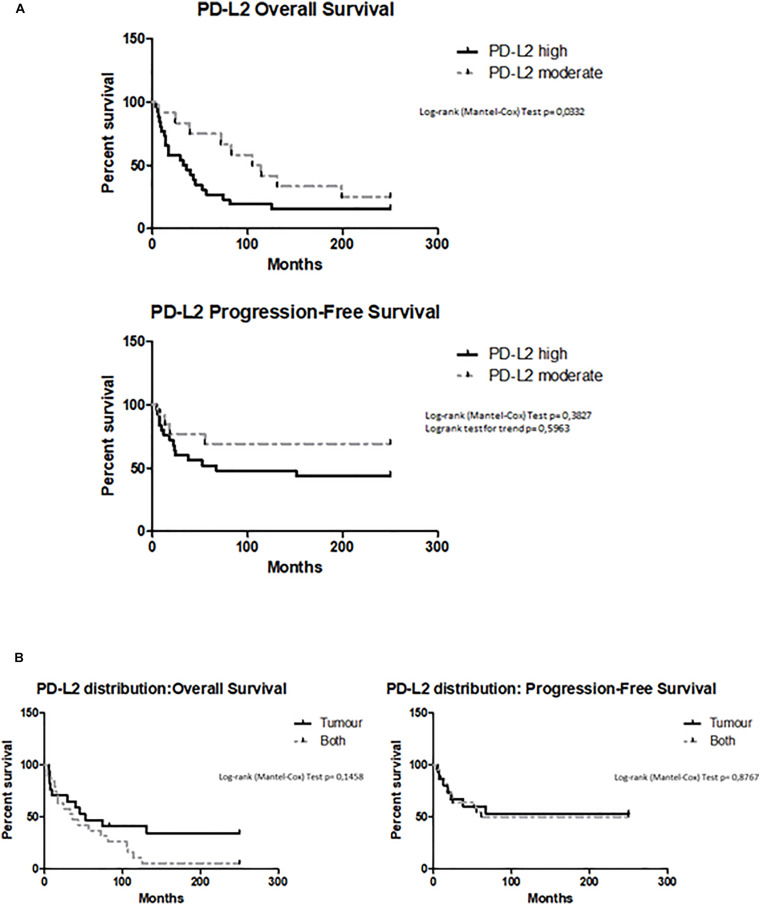
**(A)** Survival of EC patients according to PD-L2 expression. Kaplan–Meier survival curves showing overall survival (OS) and PFS of EC patients. The log-rank test with corresponding *p*-values applies to the PD-L2^*high*^ and PD-L2^*moderate*^ curves. **(B)** Survival of EC patients according to the PD-L2 distribution. Kaplan–Meier survival curves showing OS and PFS of EC patients, according PD-L2 distribution inside tumoral mass (tumor, stroma, or both). The log-rank test with corresponding *p*-values applies to the PD-L2–tumor and PD-L2–both.

Overall survival (OS) and PFS were evaluated by Kaplan–Meier analysis stratifying patients according to high and moderate PD-L2 expression levels. The subgroup with low expression was excluded because there were too few patients for a statistical analysis. Kaplan–Meier analysis revealed that OS was significantly longer for patients who have a lower PD-L2 expression (PD-L2^*high*^ 34 months vs PD-L2^*moderate*^ 114 months, *p* = 0.0332, HR = 2.033, 95% CI = 0.9747 to 4.240) (Figure 4A). Significant correlation with better prognosis was confirmed for patients with moderate expression of PD-L2 also with the Cox proportional hazards model, adjusted for age (Pr > χ^2^ = 0.029, HR = 0.26). For PFS, PD-L2 expression has minor impact on progression-free status (*p* > 0.05, HR = 1.942, 95% CI = 0.7419 to 5.085) (Figure 4A). Additionally, OS and PFS were calculated according to PD-L2 distribution, dividing patients for PD-L2 expression in tumor, stroma, or both. The stroma subgroup was excluded because there were too few patients for a statistical analysis. PD-L2 distribution inside tumoral mass does not influence OS outcome (*p* > 0.05, HR = 0.5368, 95% CI = 0.2550 to 1.130) or PFS (*p* > 0.05, HR = 0.9280, 95% CI = 0.3610 to 2.386) (Figure 4B).

### PD-L2 Expression Stimulates Migration and Survival of EC Cells

**FIGURE 5:**
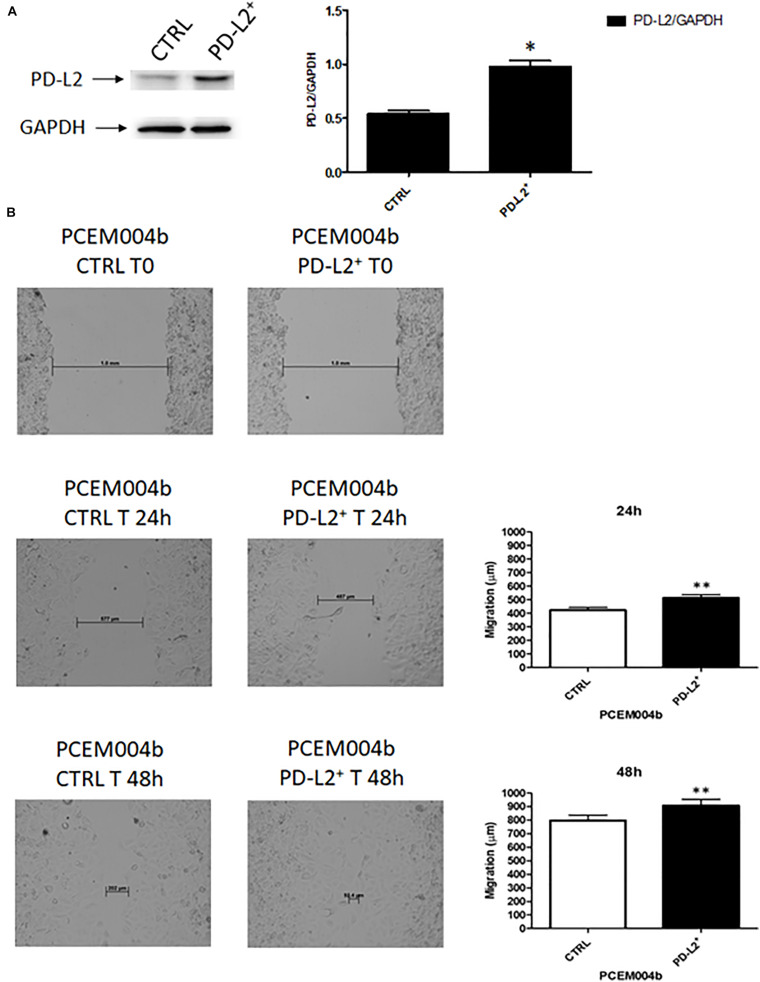
PD-L2 overexpression enhances migration of EC cells. **(A)** Western blot analysis of PD-L2 and GAPDH protein levels in PD-L2^+^ PCEM004b cells. Blots are representative of one of three separate experiments. PD-L2 densitometry values were normalized to GAPDH used as loading control. Densitometric values shown are the mean ± SE of three separate experiments. *p < 0.05 vs control cells. **(B)** Wound healing assays for PCEM004b cells after PD-L2 overexpression. All experiments were repeated three times. T0 h (used for consistency with other time-points). Data are presented as the mean ± SD. **p < 0.01 vs control.

**FIGURE 6:**
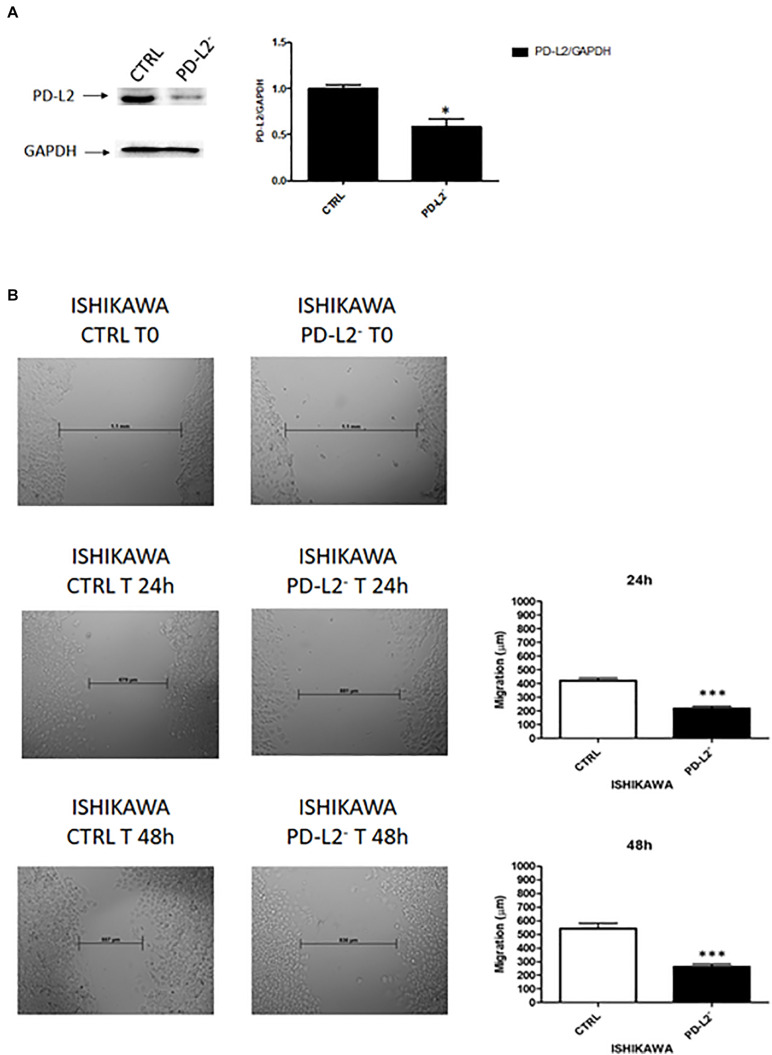
**(A)** PD-L2 silencing reduces migration in Ishikawa cells. **(A)** Western blot analysis of PD-L2 and GAPDH protein levels in PD-L2–Ishikawa cells. Blots are representative of one of three separate experiments. PD-L2 densitometry values were normalized to GAPDH used as loading control. Densitometric values shown are the mean ± SE of three separate experiments. *p < 0.05 vs control cells. **(B)** Wound healing assays for Ishikawa cells after PD-L2 silencing. All experiments were repeated three times. T0 h (used for consistency with other time-points). Data are presented as the mean ± SD. ***p < 0.001 vs control.

**FIGURE 7:**
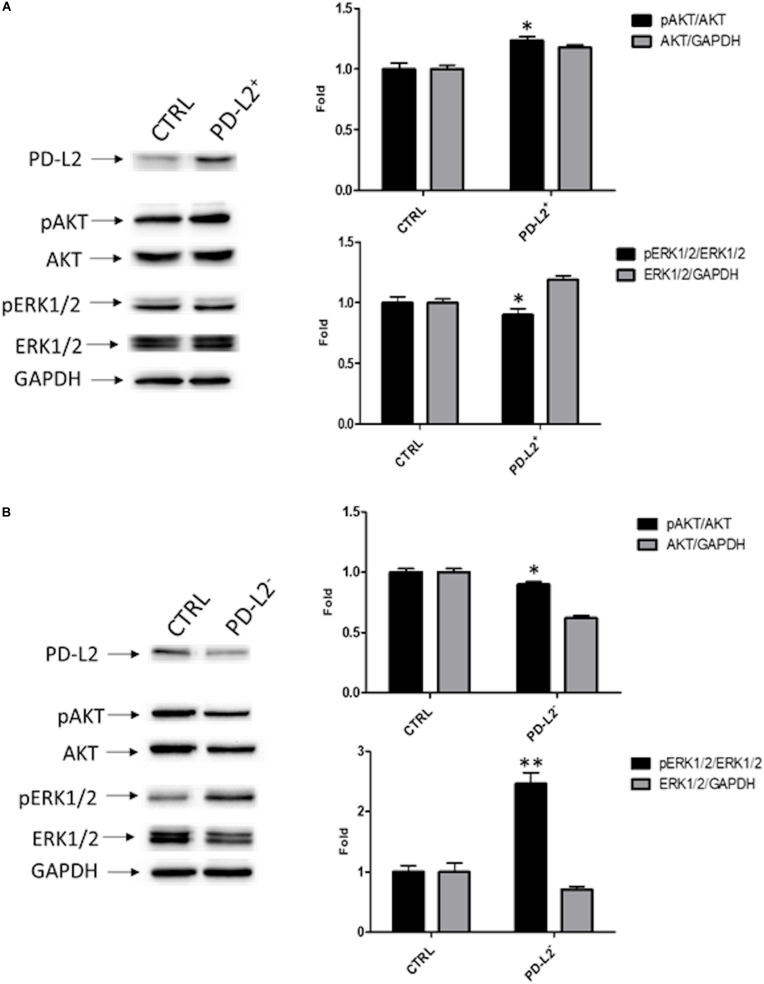
PD-L2 influences AKT and ERK pathways. **(A)** Western blot analysis of pAKT (Ser473), pERK1/2(Thr202/Tyr204), ERK1/2, AKT, and GAPDH protein levels in PD-L2^+^ EC cells. **(B)** Western blot analysis of pAKT (Ser473), pERK1/2(Thr202/Tyr204), ERK1/2, AKT, and GAPDH protein levels in PD-L2^–^ EC cells. Blots are representative of one of three separate experiments. The pERK1/2(Thr202/Tyr204) and pAKT (Ser473) protein levels were determined with respect to ERK1/2 and AKT levels. ERK1/2 and AKT densitometry values were normalized to GAPDH used as loading control. Densitometric values shown are the mean ± SE of three separate experiments. *p < 0.05 vs control cells.

To examine the role of PD-L2 in regulating migration of EC cells, the mixed type I/II PCEM004b cell line, showing a low PD-L2 expression, was transfected with a PD-L2 overexpressing vector (PD-L2^+^), whereas Ishikawa cells, which are a type I model expressing high levels of PD-L2, were silenced using a PD-L2 siRNA (PD-L2^–^). PD-L2 expression was subsequently detected by Western blot (Figures 5A, 6A). The results showed that PD-L2^+^ cells exhibited higher migratory capacities compared with the control group (empty vector-transfected cells) as determined by the wound healing assay (*p* < 0.01) (Figure 5B). Opposite results were obtained in PD-L2-silenced Ishikawa cells since PD-L2 cells exhibited lower migratory capacities compared with the control group as determined by the wound healing assay (*p* < 0.001) (Figure 6B). These data indicate that PD-L2 is involved in migration of EC cells, and it could explain the worse OS for patients with high expression of PD-L2. Additionally, to assess the potential role of PD-L2 in regulating protumoral pathways involved in cancer cell aggressiveness, the modulation of ERK and Akt/PKB pathways were evaluated through Western blot analysis in PD-L2^+^ and PD-L2^–^ cells. Indeed, the phosphorylated form of AKT is significantly increased (*p* = 0.05), while phosphorylated ERK is significantly decreased in PD-L2^+^ cells, compared to the control (*p* < 0.05) (Figure 7A), and the opposite effect was observed by PD-L2 silencing in Ishikawa cells (Figure 7B).

### PD-L2 Expression Influences Chemotherapy Response in EC Cell Lines

**FIGURE 8:**
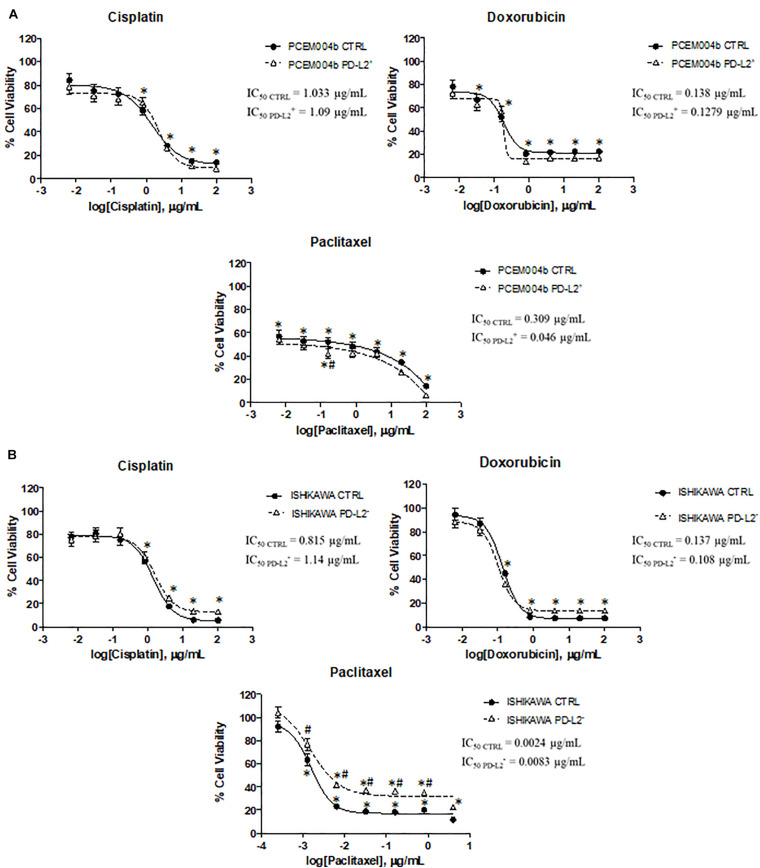
PD-L2 influences chemoresistance in EC cell lines. **(A)** PD-L2^+^ and **(B)** PD-L2^–^ cell lines and relative controls were treated for 72 h with different concentrations of cisplatin, doxorubicin, and paclitaxel (up to 100 μg/ml). Data shown are expressed as mean ± SE of three separate experiments. *p < 0.05 treated vs vehicle, #*p* < 0.05 PD-L2^+^ or PD-L2^–^ vs control.

To evaluate the potential role of PD-L2 in influencing response to chemotherapy, PD-L2^+^ and PD-L2^–^ EC cell lines were treated with the common chemotherapeutic drugs used in EC therapy (cisplatin, doxorubicin, and paclitaxel) for 72 h, and cell viability was evaluated compared to the respective control. The results showed that PD-L2 does not significantly alter the sensitivity of both transfected/silenced cells to cisplatin and doxorubicin (*p* > 0.05) but increased the effect of paclitaxel (*p* < 0.05) in PD-L2^+^ cells, especially at concentration of 0.16 μg/ml and decreased the effect of paclitaxel in PD-L2^–^ cells, up to 0.8 μg/ml (*p* < 0.05) (Figure 8).

## Discussion

EC is still the most frequently diagnosed malignancy of the female genital tract, particularly in developed countries (19). Type II EC is responsible for most EC-related deaths because it is characterized by an aggressive behavior, late stage detection, and high resistance to common therapy. Furthermore, there are no specific targeted therapies for this subtype, and it is still treated in the same way as endometrioid type I EC, which is characterized by good prognosis and good response to therapy. Therefore, type II EC needs new treatment options and molecular targets (20). PD-L1 and PD-L2, as PD-1 ligands, are considered signals that negatively regulate T-cell activation, stimulating tumor immune escape and survival (21), but emerging evidence shows that they also have tumor-intrinsic functions (13, 15, 22). The role of immune checkpoints in the suppression of T-cell antitumor response leads to the development of immune checkpoint inhibitors for cancer treatment. Indeed, blocking antibodies against PD-1 (such as Nivolumab and Pembrolizumab) and against PD-L1 (such as Atezolizumab) have been approved by the US Food and Drug Administration (FDA) for melanoma, non-small-cell lung cancers and MSI tumors (8). Regarding PD-L2, less is known about its role in cancer, and up to now, there is little information available in EC, especially for non-endometrioid EC (9).

PD-L2 expression was evaluated in type II ECs in 12 patients and 7 (58,3%) were positive for PD-L2 without a significant difference among patients with different ages, differentiation status, clinical stages, histological types, or status of vascular invasion in the tumor (23). Additionally, as reported by Sung and collaborators, analyzing 127 EC tumor specimens (113 classified as type I), PD-L2 expression seems to differ from PD-L1 with PD-L1 positive staining in tumor cells at 36.2% and 64.4% with PD-L2 (24). In previous works, no significant differences in PD-L2 expression have been found between normal endometrium and type I tumor (23, 25, 26). High PD-L2 expression was shown in 30% of primary EC patients and 16% of uterine sarcoma patients, demonstrating the potential of PD-L2 blockade in limited proportion of uterine cancer patients (26). Moderately, poorly differentiated and non-endometrioid EC shows a more frequent PD-L2 expression and seems to be correlated with POLE and MSI status (9, 23).

In glioma, an unfavorable prognostic effect of PD-L2 was reported (27). Moreover, in hepatocellular carcinoma, PD-L2 expression was unfavorable both for OS and DFS/PFS, but, in esophageal cancer, high PD-L2 expression implied a trend toward favorable prognosis, suggesting that PD-L2 has different effects on immune suppression among different cancer types (17). In this study, we demonstrated that ECs express both PD-1 ligands but PD-L2 seems to be expressed more frequently than its homolog PD-L1, especially in serous subtype. Indeed, we showed that PD-L2 expression is higher in the serous subtype of non-endometrioid EC (69.23% and 15.38% with high and moderate expressions, respectively) compared with other subtypes. Moreover, we observed that PD-L2 expression correlates with shorter OS (*p* = 0.0332), and high PD-L2 expression is detected predominantly in FIGO stages I–II and IV samples. Therefore, we analyzed the possible molecular mechanisms supporting the negative prognostic role of PD-L2 in EC.

Emerging evidence shows that PD-L1 and PD-L2 also activate tumor-intrinsic functions (13, 14, 22). PD-L1 regulates cancer cell resistance to apoptosis (22), cell proliferation, AKT/mTOR signaling pathway, and autophagy in ovarian cancer cells (13, 15), while *in vitro* data, describing an intrinsic role of PD-L2 in cancer cells, are scarce. A recent study demonstrates that cell-intrinsic PD-L2 signals promote invasion and metastasis through the Rhoa-ROCK-LIMK2 and positively regulate autophagy pathways in osteosarcoma cells (14). In this study, we demonstrate that PD-L2 increases migration of the mixed type I/II PC-EM004b cell line, while the opposite effects were observed by PD-L2 silencing in the Ishikawa cell line, a type I model that expresses high levels of PD-L2.

The Ras/Raf/MEK/ERK and Ras/PI3K/PTEN/Akt pathways interact with each other, regulating a variety of oncogenic processes including cell proliferation, survival, epithelial mesenchymal transition, enhanced motility, angiogenesis, and genetic alterations (28–30). Furthermore, increased PI3K/AKT/mTOR signaling has been reported in both type I and type II ECs (31). Previous results clearly suggest that PD-L1 is able to activate an intrinsic signal through the mTOR/AKT pathway, supporting cancer cell proliferation and regulating cell autophagy (13, 15), but there is no evidence for the involvement of PD-L2 in these pathways. We first demonstrated that PD-L2 overexpression induces an increase in AKT activation and a reduction in ERK phosphorylation. Indeed, the Ras–ERK and PI3K–AKT pathways can negatively regulate each other’s activity. It has been reported that this cross-inhibition is often revealed when one pathway is chemically blocked, thereby activating the other pathway (28).

Furthermore, the clinical significance of the PD-L2 expression and chemotherapy has not been fully investigated. Tanaka et al. demonstrated that esophageal patients with PD-L2-positive tumor had significantly inferior responses to chemotherapy suggesting that PD-1/PD-Ls pathway might be an immunological mechanism associated with resistance to chemotherapy in esophageal cancer patients (32). Herein, we found that PD-L2^+^ EC cells are more sensitive to paclitaxel than the control, and the opposite effect is observed in the PD-L2-silenced cells. However, PD-L2 expression levels did not alter the response to cisplatin and doxorubicin. It has been found that a specific inhibition of MEK1/2 kinase activity, associated with a decrease in phospho-ERK, enhanced the effects of nab-paclitaxel-based chemotherapy in pancreatic ductal adenocarcinoma patients (33). Similarly, elevated levels of activated ERK have been found in paclitaxel-resistant hematopoietic cells and ectopic activation of Raf induces resistance to doxorubicin and paclitaxel in breast cancer cells (34). These evidences support our results in which PD-L2 silencing, associated with an increase in pERK1/2/ERK1/2 ratio, are more resistant to paclitaxel than PD-L2^+^ cells.

PD-1 ligands are transmembrane proteins that binds PD-1 expressed on the cellular surface of activated T and B cells, monocytes, natural killer, and dendritic cells (23, 35). Recent studies showed also an intracellular distribution, suggesting that these proteins might have an unexpected function, different from what has previously been described for its membranous counterpart (13, 14, 36, 37). It has been demonstrated that cytoplasmic PD-L1 levels in SKOV3 and HO8910 ovarian cancer cell lines are high, and in SKOV3 cells, cytoplasmic PD-L1 increased cancer cell growth and migration (36). In addition to a membrane expression, cytoplasmic PD-L1 was detected also in lymphoma (37) and lung cancer (38). For PD-L2, previous studies reported an expression in both membranous and cytoplasmic compartments (14, 38, 39), and these findings were confirmed in our study where, by confocal analysis, PD-L2 was detected in the cytoplasm and plasma-membrane.

## Conclusion

In conclusion, our preliminary data suggest a prognostic role of PD-L2 in type II EC patients. High PD-L2 expression is detected in serous and mixed subtype and correlates with a shorter OS, without affecting PFS. Anyway, a more extensive study is required in order to establish if there are differences in PD-L2 expression between type II EC and the normal uterus. Indeed, in this study, we used a small cohort of normal samples because IHC protocol was incompatible with specimens rich in fat. Overall, *in vitro* investigations revealed that PD-L2 affects two main protumoral pathways in EC cells. Furthermore, it is involved in cancer cell migration and in influencing sensitivity to paclitaxel, suggesting that PD-L2 could have an additional non-immunological role supporting EC malignancy.

## Data Availability Statement

Publicly available datasets were analyzed in this study. This data can be found here: https://www.cbioportal.org/study/summary?id=ucec_tcga_pan_can_atlas_2018.

## Author Contributions

OM contributed to the acquisition, analysis, and interpretation of the data, and drafted the manuscript. DA, ST, MBM, and CA participated in the acquisition and interpretation of the data. GS revised the manuscript. FM, LZ, and CA participated in the acquisition of the data. BF and FA contributed to the conception of the work. MN designed the work and drafted the manuscript. All authors contributed to the article and approved the submitted version.

## Conflict of Interest

The authors declare that the research was conducted in the absence of any commercial or financial relationships that could be construed as a potential conflict of interest. The reviewer CF declared a shared affiliation, though no other collaboration, with one of the authors FM to the handling editor.

## Footnotes

1 http://cbioportal.org
